# Becoming and being a translation and interpreting teacher in China: A sustainable role identity trajectory

**DOI:** 10.1016/j.heliyon.2024.e36013

**Published:** 2024-08-08

**Authors:** Bacui Chen, Jing Huang

**Affiliations:** a School of Foreign Studies, Lingnan Normal University, Guangdong, China; b Department of English Language Education, The Education University of Hong Kong, China

**Keywords:** Translation and interpreting teacher, Role identity trajectory, Sustainable development, Career path

## Abstract

This study presents a Translation and Interpreting Teacher Role Identity (TITRI) trajectory, a sustainable career development model for T&I teachers. It employs a qualitative case study method to investigate the professional lives of three T&I teachers in China, mapping their role identity development across three stages using the Dynamic Systems Model of Role Identity (DSMRI). The findings highlight that the three case participants have experienced some major events, symbolizing a career path beginning as a T&I practitioner and culminating into a multifaceted role. The analysis emphasizes how major events define the TITRI trajectory, shaping T&I teachers’ professional paths and highlighting the dynamic interplay between their personal experiences and professional development. The TITRI trajectory enhances our understanding of the professional role identity of T&I teachers. It adds to the existing literature on T&I teacher professional development by providing deeper insights into the mechanisms that help T&I teachers in cultivating their roles as trainers/educators, researchers and practitioners within the higher educational context.

## 1 Introduction

Professional identity development is a crucial aspect of sustainable career development for teachers, notably for those translation and interpreting (T&I hereafter) teachers. In China, teachers who are specialized in teaching translation and interpreting in tertiary contexts are known as T&I teachers. Typically, these teachers often work in academic institutions with the goal of preparing students for future careers as translators or interpreters. Despite the substantial demand for and the large number of T&I teachers, little is known about how T&I teachers develop their professional role identity over time and how this development influences their career trajectory. Though previous studies have explored the concept of professional identity focusing on various aspects, there is still a need for a more specific and nuanced understanding of T&I teacher role identity trajectory, which takes into account challenges brought by the combination of roles and opportunities they face in their career development.

In China, T&I industry has grown impressively, with a total number of T&I professionals reaching 6.42 million by the end of 2023, an increase of 6.8 % from the previous year. Despite this rapid growth and the establishment of nearly 500 universities offering T&I degree programs (both undergraduate and graduate levels), there is still a significant shortage of qualified T&I teachers. This disparity between the rapidly increasing demand for T&I professionals and the limited number of qualified T&I teachers underlines a pressing need for sustainable professional development in the field. Given the dynamic and demanding nature of the T&I industry, a framework that not only supports long-term professional engagement but also enables the teachers to adapt to changing standards is urgently needed. The sustainable trajectory reveals how to improve T&I teachers’ job satisfaction and engagement, contributing to a more stable and rewarding professional environment.

This article reports on a conceptual model termed as T&I Teacher Role Identity (TITRI) trajectory to support this sustainable development. The TITRI trajectory is a three-stage model that charts the professional role identity development of T&I teachers with each stage characterized by major events and challenges that T&I teachers must navigate to progress along the trajectory. By using a qualitative case study research approach and examining the experiences of three T&I teachers in China, this study illuminates how understanding role identity trajectories can inform strategies to enhance career longevity and success. The results could provide insights into the sustainable career development of T&I teachers in China and the factors that influence their career trajectory. Further, this study offers practical implications for T&I teachers and policymakers, highlighting the importance of personalized professional development and tailoring policy supports to optimize career progression.

The rest of the paper starts with a review of related literature on teachers career trajectory and the idea of being and becoming of a T&I teacher, followed by the presentation of a theoretical model, termed “Dynamic Systems Model of Role Identity” (DSMRI), before presenting the case study methods and research findings to explore the key stages of T&I teachers’ sustainable development trajectory. In closing, the article outlines a TITRI Trajectory consisting of three different stages, which range from practitioner to practitioner-trainer/educator-researcher.

## 2 Career trajectories of teachers

The study is grounded in the wider context of investigations into teachers’ career trajectories. With Huberman [1], Fessler and Christensen [2], and Sikes [3] as the key contributors, teacher education research has focused heavily on the career trajectories of teachers since the 1980s. In his highly influential book *The Lives of Teachers*, Huberman [1] offered a key study of seven career phases as experienced by middle school and high school teachers in Geneva, describing the contrasts and commonalities of 160 teachers via extensive interviews. Overall, the models they provided separated the whole career trajectories of teachers from graduation to retirement into several phases or stages, with the aim of discovering a broad pattern to explain the career paths of teachers.

Aside from recognizing basic trends, individual variations also matter in the career trajectory of teachers. In this respect, Day et al. [4] undertook the most thorough, large-scale, and exhaustive analysis of teachers career trajectory variations between 2001 and 2005. The VITAE (*Variation in Teachers' Work, Lives, and Effectiveness*) project involved 300 primary teachers working in 100 schools across 7 local authorities to determine the factors that contributed to variations in teachers' effectiveness at different phases of their careers and in different school contexts. Based on an abundance of data, the VITAE defined six professional career phases according to the number of years a teacher has spent in the profession. These studies have consistently demonstrated that teachers' professional development is influenced greatly by their career stage. For example, early career stages are typically marked by high levels of commitment and the need for substantial support, while mid-career stages may involve managing increasing tensions between professional and personal lives. Further, the VITAE study introduced a more individual approach to interpret teachers' careers, suggesting that the career trajectories are less linear and more individualized [5]. This perspective, together with the previous research, support the development of more complex theories and frameworks to investigate how teachers’ career paths can be influenced by various combinations of factors [6].

While considerable studies, either small-scale case studies ([7]) or large-scale nationwide investigations [4] have outlined the career development trajectory based on a clear timeline, i.e. first, mid-career, and final years, few have been devoted to the role identity development of teachers. This conventional approach, however, overlooks how critical events like role identity changes, significant achievements, or challenges might actually drive transitions between stages. Such oversights points to the necessity for frameworks that recognize these major events as essential to understanding professional development in a more holistic way. Yet, understanding the trajectory of teacher role identity development is crucial as it significantly impacts their professional development [8] and ultimately determines what kind of teachers they become. Insights can be gained into how teachers develop their identities in different contexts [9,10], which can in turn inform teacher education and professional development programs. Additionally, studying the role identity trajectory of a specific group of teachers, especially T&I teachers, who are expected to establish a profile of a trainer/educator-practitioner-researcher [11], can help us better understand how teachers form their main role and sub-roles within specific contexts, and how these roles relate to professional disciplines [12]. Overall, researching teacher role identity trajectory can deepen our knowledge of the mechanisms that help teachers develop their professional identities and contribute to the literature on teacher professional development.

## 3 The being and becoming of a T&I teacher

Being a T&I teacher at a university is never easy. T&I teachers, like those in other practice-related fields, are required to juggle a variety of obligations and dedicate significant time to each. They must prioritize assignments and manage their time to meet the expectations of students, institutions, and translation clients, among others. As university faculty, they are first and foremost expected to be T&I trainers, with the responsibility of preparing future practitioners and making them aware of industry norms and conventions [13,14]. Other than conveying just knowledge and skills, they are also educators, accountable for educating students into well-rounded, cultural individuals with innovative mindsets. Second, because higher education institutions are academic in nature, full-time teaching staff are expected to be heavily involved in research, with promotion and incentive schemes frequently stressing rewards for research-related commitment and success [15]. A third feature, which is determined by the practice-oriented nature of the T&I field, requires those who teach to actively participate in professional T&I practice. Individuals without direct T&I knowledge are seen as “absolutely preposterous” [13] for teaching future professionals and providing them with the adaptability and flexibility required in tomorrow's T&I marketplaces.

Drawing on a role identity perspective, the being of a T&I teacher is framed as a combination of trainers/educators, researchers, and practitioners. Professional role identity refers to the set of interpretations that T&I teachers assign to themselves in relation to the variety of roles they play and the professional activities in which they engage [16]. Despite the fact that institutional contexts usually define specific roles for teachers, T&I teachers actively shape these roles by adjusting and negotiating them based on their personal experiences and career aspirations. This allows them to work beyond institutional confines. This process demonstrates the dynamic and fluid nature of professional role identity in university setting. Instead of the commonly used term “professional identity,” the idea of “professional role identity” is employed since it stresses more the expectations imposed on teachers and the teachers’ own interpretations of roles [17]. It encompasses both the sets of expectations that are imposed on teachers in relation to these roles, and their individual interpretations and internalization of those expectations. In other words, the being of T&I teachers comprises how teachers internalize expectations related to the performance of trainer/educator, researcher, and practitioner identities, as well as how they navigate their unique combination of those identities.

Becoming a teacher is a process, replete with unpredictability, and negotiations, requiring the acquisition of new professional knowledge and understanding [18]. Given the constant construction, reconstruction, and expansion of T&I teacher identities, the emphasis on time and place of learning becomes critical [19]. Viewed through the lens of role identity, the current role identity of T&I teachers may stem from and be impacted by a previous T&I student role identity and be shaped by future envisioned role identity. Initially, T&I teachers may primarily engage in T&I practice or training/educating tasks, assuming the roles of T&I practitioners and trainers/educators. As their careers progress, they may increasingly devote time to T&I research to fulfill institutional promotion requirements, adopting the role of researchers. This interplay of roles can culminate in the formation of numerous, interacting role identities, which in turn shape the development of T&I teachers.

Thus, the sustainable career trajectory of T&I teachers requires an understanding of their multiple role identities. Sustainable development in this field involves a long-term commitment to continuous growth, development, and improvement, resulting in sustained and enhanced achievements [20]. T&I teachers can achieve sustainable development by integrating their roles as trainers/educators, researchers, and practitioners, advancing their careers through personal initiatives [21]. This ongoing process requires acquiring new knowledge and skills to meet changing needs and obstacles, enabling T&I teachers to adapt to new circumstances and stay competitive in the field. They also contribute to the process of T&I knowledge and practice through their teaching and research while maintaining close links to their T&I practice. In essence, the sustainable development of T&I teachers involves creating conditions for them to embody the roles of trainer/educator-practitioner-researcher and fostering their growth in order to develop such a multifaceted profile [11]. Therefore, the current study aims to document the role identity trajectory of T&I teachers to ensure the sustainability and success of their career development in a practice-related discipline.

## 4 Dynamic systems model of role identity (DSMRI)

The present study was guided by an identity viewpoint, based on the Dynamic Systems Model of Role Identity (DSMRI) [22]. In an effort to guarantee the sustainability of role identity development, the DSMRI uncovered characteristics that link the past, present, and future role identities. In the systems approach, the temporal dimension includes not just the teacher's current role identity, but also the teacher's past and predicted future roles [23].

**Fig. 1:**
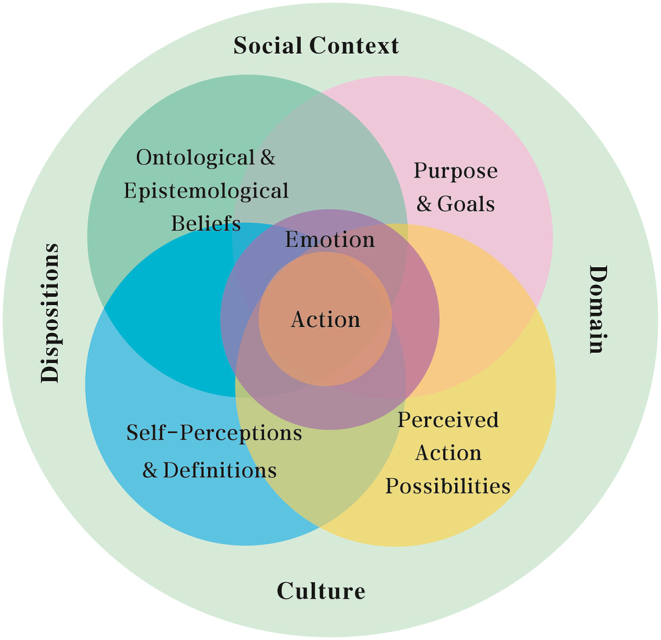
The dynamic systems model of role identity (DSMRI) [22].

DSMRI [22,23] is used to comprehend the rich, dynamic, contextualized, and multifaceted character of identity phenomena. It utilizes role identity as the fundamental analytical unit and role as a significant organizational framework for T&I teachers' narratives about their history, present, and future (Fig. 1). Role identity is shown by the DSMRI as including four components that are positioned in a social context and mediated by cultural means. These components are dynamic and mutually influential, and they are subject to continuous change. The first component is T&I teachers' ontological and epistemological beliefs, i.e., their knowledge and ideas about the world or the T&I domain, including pedagogy, research, and practice, that are proclaimed as being true. For instance, a T&I teacher may strongly feel that a PhD is a necessary for career progression, whereas other teachers may disagree. The second component is T&I teachers’ self-perceptions and self-definitions, which include components of the T&I teacher within a specific role, such as self-defined qualities, group affiliations, and how these are viewed to relate to their role(s). A T&I teacher may see himself/herself as inquisitive, industrious, or devoted to specific values. It also involves self-recognition as a member of an academic group, such as the T&I research or teaching community. The third component is the purpose and aims of T&I teachers, encompassing broad and particular, long-term and short-term goals for their teaching, research, and practice. The aims of T&I teachers might vary along a variety of dimensions spanning multiple roles, and eventually lead to sustainable and successful professional advancement. The fourth component is perceived action possibilities, which are the perceptions, intents, strategies, and actions that T&I teachers regard as possible or impossible in light of their beliefs, self-perceptions, and definitions in order to attain their objectives. For instance, a T&I teacher may see the integration of three responsibilities as a significant step toward professional progress, but may not perceive the integration as action possibilities due to limited time and energy.

Simultaneously, these four components interact in a dynamic manner, emerging through social interactions in different contexts. The degree of alignment characterizes the relationship between the interdependent components. These four components interact to establish the foundation for decision making and action in a particular role in a specific situation [24].

The complexity grows when multiple role identities are included. Because the role identity system of T&I teachers encompasses the sub-systems of trainer/educator, researcher, and practitioner, each role identity system serves as a role of the DSMRI system for T&I teachers, each of which is considered as comprising the four interdependent components described above [22]. The T&I teacher role identity system is the result of the integration of numerous role identity systems. A T&I teacher may hold certain beliefs about T&I training, such as how important it is to students’ T&I proficiency, or about T&I practitioners, such as how well-paid they are in the market. These beliefs will be consistent with their professional goals of working as a T&I trainer/educator and practitioner. A T&I teacher may also be processing a self-perception as a researcher trying to overcome obstacles in order to fulfill institutional demands. His/her action options may include joining a research community to share her knowledge and research abilities, or applying for a research grant to obtain authorization to conduct a higher level research project. Taking actions as a T&I practitioner may have an influence on his/her ideas about how and why others might learn T&I, modifying his/her action possibilities of becoming a T&I trainer/educator and possibly pursuing integration as a future career goal.

## 5 The past, present, and future of role identity

**Fig. 2:**
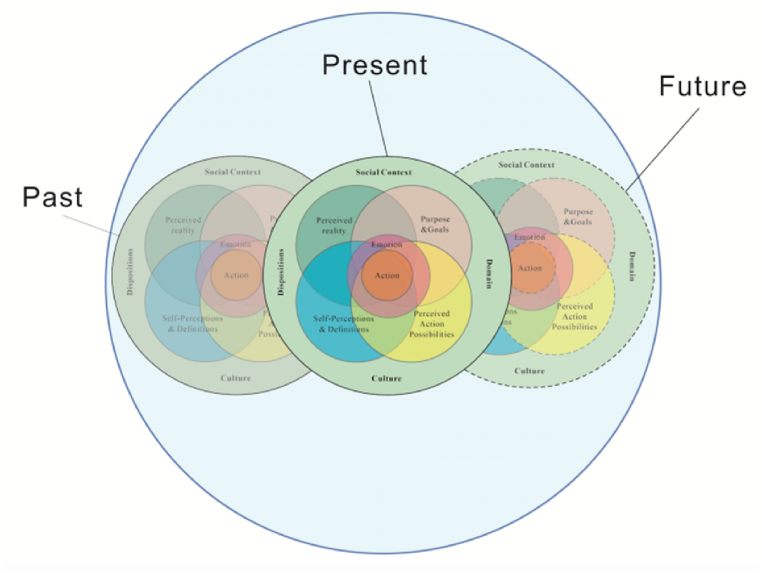
Past, present, and future of role identities (adapted from Kaplan and Garner [22]).

The fact that the DSMRI integrates a temporal component is visible in the past, present, and future dimensions of the role identity system [22,25]. The DSMRI system comprises past role identities that were inhabited before or are no longer occupied today, as well as role identities for future roles, in addition to the role identities that a T&I teacher now occupies. A present T&I teacher's trainer/educator role identities encompass a T&I student's past role identities as well as perhaps a T&I researcher's future role identities (Fig. 2).

Past and future role identities are related to the present in that they give content and structural foundations for the formation of present role identities [22]. For example, in Garner and Kaplan's study, Pat's present role identity as a physics teacher is derived from and influenced by his past role identities as a high school and college student and a student-teacher in the way that his self-perception and definition as a hard-working but not particularly talented learner of physics and an inspiring teacher [26]. Huang et al. [27] observed three novice teachers' beliefs changing as a result of their previous role identities, which impacted their self-perception and definition. Courtney's self-related purpose and aspirations were provided by a then-future-imagined role identity of a teacher inside her present role identity of a practicum student (e.g., to motivate the class and to expose them to English). Bao and Feng [28] associated a positive future researcher role with contextually relevant action possibilities for achieving a goal, while emphasizing the difficulty of such achievement and promoting congruence between future-imagined possible roles and their present college English teacher identity. The domestic visiting study program (a visiting scholar program within mainland Chinese universities) plays a crucial role in helping college English teachers who identify themselves primarily as teaching staff to develop their research capacity and transition into the researcher role.

Utilizing the DSMRI model, “major events” in the study are defined as significant occurrences that prompt notable shifts or transformations on one or more of the four components of a T&I teacher's role identity system. For example, taking on new roles such as researcher or practitioner, or encountering changes in institutional requirements can trigger a teacher to reevaluate and readjust their role identities. A “stage” in DSMRI language is a distinct phase in career trajectory of a T&I teacher, characterized by a relatively stable interaction among the four DSMRI components, leading to a harmonious state among role identities. Each stage corresponds to a certain period in T&I teacher career path that remains stable until disrupted by major events that transition them to a new stage. “Sustainable” refers to a continuous process in which a T&I teacher dynamically integrates and adjusts his DSMRI components. This guarantees that T&I teachers are capable of adjusting their professional roles, responding to institutional demands and their own development, making them both resilient and effective throughout their professional careers. This DSMRI framework, together with mapping out major events and stages, provides a systematic approach to understanding and facilitating career development. It clarifies the progression through distinct phases, making it easier to analyze and describe the development of role identities. This study is pioneering in its use of DSMRI to explore T&I teachers' role identities across their past, present, and future, and it is the first to apply the model to the concept of a sustainable teacher career trajectory, thereby enriching the study with a structured method to analyze the dynamic interplay of professional role identities.

## 6 The study

The study employs a qualitative case study design to investigate the role identity trajectory of three T&I teachers in mainland Chinese universities. Data sources included three rounds of in-depth interviews with the three participants, complemented by one field trip observation. In addition, other forms of interaction, such as social media exchanges, and participants’ published autobiographies served to provide invaluable insights into the experiences of these T&I teachers. The present study aims to answer the following two research questions:

(1) What are the three T&I teachers' role identity trajectories?
(2) Are there any major events in their role identity development? If yes, how do these events shape their role identity trajectories?

The present study used the qualitative case study method as it allows for an in-depth investigation of a phenomenon within its real-life context — particularly useful when aiming to explain “how” and “why” something happened [29]. As this study attempts to unearth T&I teacher role identity trajectory and the factors affecting its development, the case study approach permits a comprehensive examination of these intricate and dynamic processes in detail through the analysis of individual cases. Additionally, the explanatory case study adopted in the present study, is well suited to examine the underlying mechanisms behind the phenomenon being studied [29]. By purposefully selecting three cases, the study aims to provide a detailed investigation of each case in order to understand the mechanism behind their trajectory.

The study uses analytic generalization to explore unique case studies, rather than aiming for broad statistical or empirical generalizations [29]. It employs DSMRI as the guiding framework to explore the distinct and intricate identity trajectories of individual T&I teacher in China. Each case is carefully analyzed to bring out its unique characteristics and complexities, showcasing the varied experiences of these T&I teachers. The findings emphasize the particularities of each teacher's career journey, illustrating how these specific insights deepen our understanding of professional identity development in the field. While the insights drawn from each case study are believed to be profound, they are contextual and not intended to be extrapolated universally to other scenarios.

### 6.1 Research setting and participants

For this study, a purposefully diverse sample of in-service T&I teachers at mainland Chinese universities with expertise as trainers/educators, researchers, and practitioners was selected. The selection criteria ensured that participants were comfortable sharing their experiences in interviews, were able to devote adequate time to the researchers and had no concerns about potential overexposure. The selected participants represented a diverse cross-section of T&I teachers, both male and female, aged between 20 and 50. They came from varying educational backgrounds and had a range of teaching experience. They worked in both Tier 1 and Tier 2 universities in Chinese mainland. Out of the potential participants located, three cases were chosen for the study, with a fine-tuned focus on T&I teachers' role identity trajectory. The chosen cases were selected based on their ability to clearly articulate their career trajectories, ranging from past experiences to present situation and future aspirations. Additionally, their diversity in backgrounds and experiences within different institutional settings provided ample scope for extending the trajectory's applicability in the future. Table 1 provides a summary of case participants' background information.

**Table 1:** A list of participants (pseudonyms).

Name	Gender	Years of being a T&I teacher	Professional title	Highest educational qualification	Types of university	Working language pair
Amy	Female	7	Lecturer	MTI	Tier 2	Chinese-English
Dina	Female	15	Associate professor	PhD in English language and literature	Tier 1	Chinese-English
Keven	Male	22	Professor	PhD in Translation Studies	Tier 1	Chinese-English

### 6.2 Data collection and analysis

The data for this study were primarily collected through in-depth interviews conducted online due to Covid-19 restrictions. Initially, face-to-face interviews and observations were planned. However, campus lockdowns and travel restrictions forced a switch to virtual platforms, such as Tencent Meeting or WeChat. Unfortunately, most of the observations had to be canceled. However, one exception was a field trip undertaken by the first author to Keven's place of work, which happened several days right before the Covid lockdown. Despite these disruptions, the Covid-19 situation had some unexpected benefits for both researchers and participants. Researchers were able to include more T&I teacher participants from different geographical locations across the country without incurring significant travel costs. For the participants, the lockdown gave them relatively comfortable “work-from-home” settings in which they could be interviewed at their convenience. A somewhat comfortable space was set aside for them to reflect on and discuss their past histories, present experiences, and future expectations [30].

Three rounds of interviews were conducted with each participant respectively within one academic semester, with each interview lasting about 1 hour. Prior to these interviews, informed consent was obtained from each participant. Given the unique and difficult circumstances imposed by Covid-19 pandemic, as well as the potentially demanding schedules of the participants, flexibility for rescheduling interviews was provided. This allowed participants pauses and resume the three rounds of the interviews at their convenience within the semester, rather than being obliged to be interviewed within certain spaced intervals.

The first round of interviews focused on participants’ background and decisions to be T&I teachers. We aimed to have a basic idea about their personal and professional histories, the experiences that led them to choose a T&I related career, and the goals that motivated them in their early careers. Prompts such as “What inspired you to become a T&I teacher?” and “Tell me about your academic experience.” were used to develop rapport and stimulate their recall of their memories.

Roughly one month after the first interview, the second round of interviews took place, concentrating on the participants’ career trajectories. In this round of the interviews, we invited participants to narrate their career trajectory experiences, including highlights, obstacles and dilemmas, and events or experiences that were meaningful to them related to each role. We were particularly interested in uncovering their pivotal moments, phases of professional growth, and instances of struggles throughout their careers as T&I trainers/educators, researchers, and practitioners. Prompts included “What were the most memorable experiences you had as a T&I trainer/educator, researcher, and practitioner, respectively?” and “What were your career hurdles or problems, and how do they affect your attitude toward becoming a T&I teacher?”

The last round of the interviews started around a month after the second round. Participants first had the chance to reflect upon and add any additional facts or thoughts to their previous interview responses. This was done to make sure that any new insights that could have emerged with time could be incorporated into their narratives. We then turned our attention toward the participants’ future plans and goals in training/educating, research, and practice as T&I teachers. Our objective was to comprehend their vision for professional career, what obstacles they expected to face, and how they planned to overcome them. To facilitate this exploration, prompts such as “Are there any plans for future training/education, research, and practice, respectively? If yes, what are they?” and “Is there any career goal as a T&I teacher?” were used.

An in-depth examination of the participants' main role and sub-role identities, including their motivations, experiences, aspirations, and plans, was made possible by the triadic interview structure, which spaced out with roughly a month's interval between each round. Although a basic timeline was designed, flexibility was also incorporated to provide for unexpected delays or interruptions, ensuring that participants had enough time and comfort to share their experiences. All the interviews were audio-recorded and transcribed manually to capture the essence and content of the discussions. Identifying names and institutions were removed, and pseudonyms were assigned to maintain confidentiality.

In addition to these three rounds of semi-structured interviews, this study also included social media contacts, particularly through WeChat Moments, and a single field visit to one of the case participant's work place. For instance, from WeChat Moments, we learned significant events like Amy's withdrawal from doctoral program, Dina's publication of a new SCI article. Furthermore, we saw firsthand the high demand for staff research that was made explicit on the noticeboard during a field trip to Keven's work place. The inclusion of these components not only improved our comprehension of the participants' real-world experiences, but was also important in our narrative construction of case participants' stories, indicating how they constructed their professional role identities in the changing context of Chinese higher education during the Covid-19 pandemic.

The ultimate objective of data analysis is to “retell” the stories using DSMRI language and structure. The data analysis process utilized DSMRI as a framework for structuring and interpreting the data. This process entailed a systematic examination of each interview, focusing on DSMRI core components: beliefs (both ontological and epistemological), self-perceptions, purpose and goals, and perceived action possibilities. A coding scheme was developed, defining roles (T&I trainer/educator, researcher, and practitioner) and their four corresponding components as the primary unit of analysis [26]. This scheme facilitated data categorization, ensuring a comprehensive investigation of each aspect of every role identity. A summary was written for each role identity for each participant that includes its content, structure, and process. These summaries laid the foundation for identifying major stages in role identity development, interpreted in light of the study's research questions. Integrating these components into narrative reconstructions allowed for the retelling of T&I teachers' stories through the DSMRI lens, reflecting dynamic interplay of these dimensions.

In the process of retelling these stories, identifying these stages involved analyzing how participants described their experiences of embracing new roles and confronting challenges—key moments that shaped their professional development. Each identified stage represented not just a point in time but a critical period of growth and transformation, underscoring the dynamic nature of their identity development.

To ensure the validity and thoroughness of the data analysis process, several measures have been taken. After developing the coding scheme, the first and second authors independently analyzed the transcripts and assigned codes to extract themes and key concepts. Inter-rater reliability was assessed, and discrepancies were resolved through discussion, deliberation, and consensus. Member check, involving sharing initial analysis and interpretations with participants and seeking their feedback and validation, was also used to mitigate biases and enhance credibility.

## 7 Findings

The findings of the study unfold through stories that capture the thematic patterns drawn from the data. This narrative approach is employed to outline the significant life and career events that the participants reported. These stories were written first to anchor the analysis around key themes, capturing role transitions and events that respond to the research questions. Each story aims to unveil a detailed picture of how specific major events influence role identity development, responding to the second research question. These narratives detail the impact of each event, providing insights into the complex dynamics of professional development and identity transformation among T&I teachers.

### 7.1 Amy's story

Amy, a passionate and dedicated individual, embarked on her doctoral journey after gaining teaching experience as a full-time T&I teacher in Chinese mainland for four years. Prior to that, she had earned her MTI (Master in Translation and Interpreting) degree in her hometown province.

Amy talked positively about her T&I practicing experience, which began while she was a T&I student. “My zeal for T&I is why I pursued a T&I teacher post,” she said, despite having “no previous experience in teaching.” This demonstrated an alignment between her personal and professional objectives, as well as her self-perception of the job. She began to establish a T&I trainer/educator role identity after being employed as a T&I teacher and soon discovered it was “different from expectation.” She cited an example of how she presumed her job as a T&I teacher was “free and unrestrained,” but in fact, “you may even have to stay up until midnight busy preparing the lectures.” Amy's early career role identity as a trainer/educator also included her self-perception of overestimating students' linguistic competence and encyclopedic knowledge, which led to her frustration and discomfort. With a hint of regret and criticism, she candidly remarked, “The students of today, I am afraid, do not reach the level of linguistic proficiency we upheld in our time.” Despite momentary failures, she considered her work as a trainer/educator “very rewarding,” especially when students were admitted into MTI programs under her supervision.

Amy's crisis as a T&I researcher was not only a result of the challenges and difficulties she faced, but also stemmed from her own self-perception and expectations. According to Amy, “I never expected it to be that difficult. I was used to being successful and this new role of researcher just didn't come naturally to me.” She was formerly recognized as an outstanding graduate student and naturally saw herself as a skilled college teacher. However, things turned out that the past action possibilities that served her well did not produce the same level of success and achievement in the new role of a T&I researcher. Her ontological and epistemological beliefs were challenged, resulting in intense feelings of doubt, hesitation, and even anguish.

Feeling stuck and overwhelmed, Amy decided to pursue further academic action possibilities to try and break through the impasse. Unfortunately, her doctoral studies in Hong Kong were marred by the 2019 Hong Kong social unrest and the 2020 COVID-19 epidemic, which nearly forced her to discontinue her studies. Reflecting on this experience, Amy admitted, “it was just one unexpected event after another. I felt upset and confused, and it seemed like continuing my studies was just impossible.” This “upset and confused” experience in her doctoral studies did not alter her pre-existing and fundamental ideas about conducting research. Consequently, she maintained a negative self-perception of her research capacity, which ultimately eroded her self-confidence in her main role as a T&I teacher. Amy's choice to leave her doctoral program at the end and return to her previous university was a pivotal moment in her career. This decision, driven by a reevaluation of her career goals and self-perceptions, is a clear example of how DSMRI components work together to influence the manifestation of T&I teacher identity. It shows how external factors, alongside personal reflection, profoundly influence one's professional identity, underscoring the complex interplay that shapes a teacher's career path.

### 7.2 Dina's story

Dina is a teacher and an alumna of a top-ranking comprehensive university. She articulated personal reasons to pursue a teaching post, with a clear career goal of integrating three sub-roles and aiming for achievement in each. At the beginning of her teaching career, she was a college English teacher and also a T&I teacher. She became a T&I simultaneous interpreter during her MA studies, and she reported having developed confidence and understanding in the role of T&I practitioner since then. Dina expressed excitement and pleasure in her T&I practitioner position, calling it “fabulous.” She recalled the experience as a practitioner as exceptionally wonderful, noting that one would “deeply miss the feeling of interpreting if they leave the profession for a while”. Her ambition to become an accomplished T&I practitioner, along with her opportunities to secure T&I practice tasks, earn a high salary, and build a reputation in the industry, well align with her belief that T&I teachers (who are also practitioners) are valued in the T&I market. Additionally, the fact that translators and interpreters are well-rewarded and respected in the market strengthens this belief. Consequently, this role identity is associated with a sense of enthusiasm.

However, Dina subsequently perceived the T&I market to be shrinking as a result of the COVID-19 pandemic. Dina's concern for her students' professional future stemmed from a conflict between her beliefs about the declining prospects for professional interpreters and her goal of preparing students to thrive in the market. Dina pondered, “if even the best students cannot secure fulfilling jobs in the T&I market, what is the purpose of our training?” This tension, while not impeding her ability to educate and train, reinforces her beliefs about the falling T&I market and, consequently, challenges her role identity as a T&I trainer/educator.

Dina experienced significant challenges in her role as a T&I researcher, which caused conflicts with other duties. At the time of the interview, her research plans had not been carried out successfully, and her investigation of new research ideas had not yet led to a new alignment of her role identities as both a trainer and researcher. She compared the failure in research with being rejected by a handsome boy:

“… the feeling of rejection is much harsher than being turned down by a handsome boy when you were young. The most difficult thing was that after rejection, where could you possibly go?”

She spoke frankly that the research failure was “cruel and disheartening.” A negative possible identity for her was an ineffective researcher. Dina's strong feelings of dissatisfaction and low self-efficacy confirmed her ontological belief that research was difficult while T&I training and practice were enjoyable. This proved counterproductive to her role identity as a researcher. Due to her unsuccessful publishing experiences, she lacked alternative ontological and epistemological beliefs, leaving her with no other action possibilities for pursuing new research ideas or meeting institutional standards.

Despite the tensions, Dina maintained a persistent attitude in addressing career difficulties as she remarked, “Working in such a university means you have to strive ahead […] I am too young to give up now, even if I feel a little powerless.” By the time this article was written, Dina had already made a breakthrough in research by publishing a SCI-indexed article about medical translation as a co-first author and presenting at a national interpreting conference.

### 7.3 Keven's story

Keven's career trajectory from an EFL teacher to a renowned T&I trainer/educator, research and practitioner paints a vivid picture of adaptability, dedication, and academic curiosity. After completing his MA, Keven began his career as an EFL (English as a Foreign Language) teacher. Later, when T&I degree programs were introduced at his institution, he was “persuaded” to become a T&I teacher based only on his “fluent spoken English.” Fortunately, instead of feeling negatively about this hasty decision, Keven embraced it as an opportunity, stating optimistically, “I was intrigued by the field of T&I though I did not know much at the beginning”. This surge of interest opens the door for his career trajectory as a T&I teacher.

After becoming a T&I teacher, he had the opportunity to comprehend and explore the action possibilities of engaging in T&I practice and research. He linked his goal of improving students’ T&I competency with his action possibilities of conducting T&I practice grounded in his epistemological certainty about his ontological beliefs that the T&I course should be deeply rooted in real-world field experience. “Theoretical knowledge is essential,” Keven often said to students, “but the true essence of T&I lies in practical application.” Later, he saw the potential of T&I research by being accepted into a PhD program at the university where he worked at that time, which provided him with more options as he developed his grasp of T&I training/education and practice.

Keven was promoted to professor and transferred to a prestigious comprehensive university after about 20 years of teaching. He is currently an established trainer/educator, researcher, and practitioner in the field of T&I. He has become a doctorate supervisor and has supervised several PhD students as a T&I trainer/educator. He has published widely in SSCI-indexed and CSSCI-indexed journals as a T&I researcher. He actively engaged in T&I practice as a T&I practitioner and was admitted as an AIIC (The International Association of Conference Interpreters) member, an international organization widely acknowledged as a symbol of high-level professionalism [31].

Keven adapted to his new work context, which is more focused on research than the formerly language-oriented university context. For Keven, his self-recognition of being a full professor and a doctoral supervisor reinforces his self-regulated goal [13] of achieving a research breakthrough to establish his value in a research-oriented university. He commented on the demanding nature of the university's requirements, saying, “it is natural expectation for universities to set such high standards, and I am committed to striving for breakthroughs.”

Keven's professional objectives of attaining the highest academic standards and his ontological beliefs motivate and facilitate his identification of additional action opportunities in his role as a researcher. Keven's story serves as a testament to the importance of adaptability, curiosity, and commitment in shaping one's career trajectory and role identity as a T&I teacher.

### 7.4 Major events in being and becoming T&I teachers

In all three cases (Amy, Dina, and Kaven), the T&I practitioner experience was consistently observed at the very early stage of their T&I career trajectory, typically when they worked as part-time translators or interpreters. However, this practitioner experience was not limited to just this phase of their career development. Their interest and involvement in T&I practice were evident prior to and throughout their journey as T&I teachers, ultimately shaping their role identities as T&I trainers/educators and researchers. Working in a university as a T&I teacher was a pivotal moment in their development of role identity among these three roles (practitioner, trainer/educator, and researcher). This milestone allowed them not only to establish a T&I trainer/educator role identity after passing the threshold, but also understand the relevance of the T&I practitioner role identity with their trainer/educator role. The expansion of the T&I researcher role, often driven by the requirements of the institutions where they served, often occurred concurrently with their main roles as T&I teachers. The majority of T&I teacher participants reported gaining research-related skills and knowledge during their PhD studies, including the ability to select a research topic, review relevant literature, write a research report, and present at an academic conference. If all went well, this was the moment for some participants, such as Keven and Dina, to enhance confidence and receive recognition from their researcher role, which was frequently associated with one or more successful experiences. In contrast, Amy experienced a sense of frustration during this period, which was associated with her research failures.

Though different T&I teachers constructed their role identities in different ways, all three cases have gone through similar major events (Table 2). These major events represent sets of properties that contribute to experiences—ways of being, that inform the being and becoming of T&I teachers. These events are described as major because they are what matters to the participants and to the researchers as we have written about the cases. Being T&I teachers has always involved who they have been, who they are, and what they envision for the future. Therefore, major events of becoming a T&I teacher draw upon the moments of being a T&I teacher.

**Table 2:** Major events for being and becoming a T&I teacher.

Amy	Dina	Keven
**In the role of T&I practitioner**		
Began interpreting as early as sophomore year for an exhibition	Interpreter for a workshop during MA study	Interpreting for international meeting after becoming a T&I teacher
Displaying great interests	Calling it a “fabulous job”	Joining AIIC
**In the role of T&I trainer/educator**		
Applying for a T&I teaching post out of passion for T&I practice	Embarking career path as English teacher but focusing on T&I teaching later	Employed as T&I teacher
Beginning T&I training/educating	Feeling the need for alignment between T&I training and practice	Becoming MTI/MA and PhD supervisor
**In the role of T&I researcher**		
Facing pressure from university	Facing pressure from university	Getting a PhD degree
Pursuing a doctoral degree but failed	Getting a PhD degree	Fulfilling in research
Experiencing frustration and setback in research journey	Fighting for a higher research profile	Being promoted to professor

In observing the career trajectory of T&I teachers, it is evident that they have progressed from T&I practitioner roles to embodying a triad of roles. They initially identified their initial T&I interests, displaying curiosity and willingness to join in T&I practice. Their interest in T&I practice was the primary motivator behind their initial decision to pursue T&I-related job development and/or persistence in the T&I career path. Formal engagement to T&I training/education began when they were employed as T&I teachers, marking a significant milestone in their career development. After becoming T&I teachers in universities, they were immersed in the higher education context, where they observed both the institution's empowering and constraining effects. It was not until they were confronted with the pressure of research requirements that our case study participants became actively involved in T&I research. They pursued doctoral degrees in order to establish and develop a T&I researcher identity, thereby cementing their career trajectory. Upon completion of their PhD studies, they gained research capacity and were more capable to integrate the three role identities.

The above table elucidates a significant overlap in our participants’ role identity development, essentially tracing a T&I practitioner-trainer/educator-researcher path. Keven, in fact, investigated the alternative route to developing role identity and candidly described it as “unrealistic.”

“Of course, there are teachers who enjoy conducting research. However, I do not believe it is practicable in the T&I profession to be determined in identifying interests from research and leveraging them to advance one's training and practice. That appears to be nearly impossible. I think the logical approach is to identify interests from practice, develop a passion for T&I, and derive pleasure from it. Subsequently, it can serve as a catalyst for the exploration of the other two roles.”(Keven)

### 7.5 Summary

In brief, the major events revealed through case stories mark key points in the role identity development among T&I teachers. These events not only indicate important stages in their career paths but also pave the way for structuring a T&I teacher role identity trajectory. The findings illustrate how major events shape their professional role identities, intertwining personal experiences with professional growth, and sets the stages for the forthcoming exploration of the TITRI trajectory. From a DSMRI perspective, the findings demonstrate the shifts of components within the professional lives of T&I teachers. The major events that mark career transitions illustrate changes in T&I teachers’ self-perceptions and professional purpose and goals.

## 8 Discussion: a T&I teacher role identity (TITRI) trajectory

**Fig. 3:**
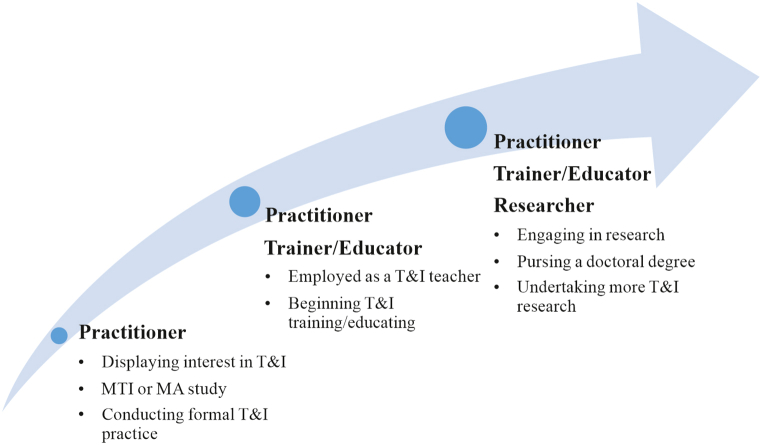
A T&I teacher role identity (TITRI) trajectory.

Reflecting on the higher education experiences of these T&I teachers from all three roles (practitioner, trainer/educator, and researcher), it becomes clear that a sustainable T&I teacher role identity trajectory requires certain important occurrences. The trajectory model delineated in this article combines these significant events into three distinct phases. It aims to expand and deepen our understanding of the mechanisms that facilitate T&I teachers in the development of professional role identities as trainers/educators, practitioners, and researchers in the context of higher education in China. The T&I Teacher Role Identity (TITRI) trajectory, which is a three-stage model of development within an upward-directed arrow, represents advancement in a specific context—in the present case, mainland Chinese universities—as illustrated in Fig. 3. The three stages of the trajectory represent significant milestones in a T&I teacher's career development path, as articulated in the findings.

In the first stage, case participants have limited access or incentive to engage in T&I training/education and research experience, but are actively engaged in T&I practice. They have not yet deliberately identified their own career path and, as a result, are not deliberately enriching their professional practice. This stage is considered as the starting point in the trajectory, highlighting the lack of awareness or sensitivity toward possible career development. Motivational resources, such as confidence, T&I training/education or research abilities, and the ongoing formation of one's future role identity in light of one's present position [32] are necessary to advance to the next stage.

The second stage is a hybrid of T&I practitioners and trainers/educators, and it necessitates active engagement in career development on the part of T&I teachers. The motivation may originate from the institutional context, as represented by external promotion requirements (Dina and Amy), or from internal identification of professional objectives for personal development (Keven). What is common for this stage is that T&I teachers acquire professional responsibilities on the job, as seen in all three cases. The participants are discovered to be looking for possibilities to participate in the Community of Practice (CoP) [33] or define their areas of research interest in the second stage. They are open to new experiences, such as learning how to teach T&I (Amy) or doing T&I research (Dina and Keven). At this trajectory in their career, they begin to connect the present with their future job prospects. To progress from the first to the second stage, the T&I teacher must make a deliberate decision to change. Changes in beliefs interplay with changes in self-perceptions and self-definitions, resulting in a rethinking of purpose and goal, and eventually in altered perceived action possibilities [34].

The second stage is essential for teachers to exercise agency in order to progress along the trajectory. The willingness to exercise agency may stem from increased confidence gained through T&I practice (Dina and Keven), enthusiasm established during the first stage (Amy and Dina), or acknowledgment felt within their university or from external sources (Amy and Dina). However, “shattered realities” [35] such as a sense of alienation from the teaching community and tension between research and other duties may have a detrimental impact on agency enactment (Dina). In fact, effective navigation through these potential tensions and the exercise of agency during this stage are perquisites for moving into third and final stage of their professional trajectory. Without such active engagement and resilience in the face of challenges, the progression into a fully-fledged T&I teacher might not be attainable.

The last stage of the trajectory is where T&I teachers engage in training/educating, research and practice in creative and productive ways. All three case participants align their career aspirations with all three roles, articulating a clear and comprehensive vision of their professional identity. This identification, clarification, and deeper awareness of their duties engages them in career development, leading to increased accomplishment and contributions to the field. T&I teachers are stated to be confident and experienced at this stage, and they may apply their expertise to new areas of research interest and development requirements. The motivation to go to the last stage might be triggered extrinsically, for example, by the imposed pressure to seek a PhD degree, produce research articles, or apply for research grants (Amy and Dina), or intrinsically prompted by the desire to explore new professional terrains (Keven). It is a step of bridging the gap between training/education, research, and practice [13] and accepting the concept of establishing a T&I trainer/educator-practitioner-researcher profile [14].

The three stages are considered integral to the process of becoming T&I teachers, as they require the development of three sub-role identities and the resolution of conflicts between the roles to integrate the roles. It illustrates the progression of from a pre-teacher to a researcher. From engagement in T&I practice to post-teacher involvement, the trajectory conceptualizes the T&I practitioner role identity, which was established prior to their formal engagement as a T&I teacher in higher education. This identity is derived from their interests in T&I practice, thereby connecting the past with the present. T&I teachers begin their professional development by investigating their T&I practice and progressing along the curve to become trainers/educators, researchers, and practitioners in higher education, therefore connecting the present with the future. Additionally, the TITRI trajectory underscores the potential for the higher education environment, as well as societal crises such as COVID-19, to both empower and entrap teachers’ role identities [36]. In the best-case scenario, as Keven illustrated in this study, they embraced their own professional development within, between, and among roles, along the trajectory, and in environments where the higher educational, institutional, and socio-cultural contexts were conducive to cultivating integration among all three roles. This resulted in high job satisfaction and optimal professional development outcomes.

Though the TITRI trajectory is grounded in the narrative accounts of participants, it is essential to acknowledge that each T&I teacher's trajectory may vary greatly. In fact, the trajectory outlined is consistent with the current demography of T&I teachers in mainland China, where the majority of teachers embark on their careers in this field after completing their master's degree [37].

However, it is critical to understand T&I teachers’ career development is neither flat nor linear [38], and teachers are not and should not be constrained to a single route. The current TITRI trajectory just serves as a conceptual model by focusing on three case participants, emphasizing the awareness of numerous roles that come into play. The model also explores how they manage conflicts and achieve harmony both between and within these roles as individuals progress toward being and becoming T&I teachers, and as they cope with conflicts, and achieve integration between and within the roles.

To achieve professional development, T&I teachers are not obligated to advance through each stage; rather, they are encouraged to establish their own path by incorporating a variety of elements in their own unique manner. In other words, the objective of TITRI trajectory is not to provide a standardized and uniform trajectory that erases personality attributes, but rather to increase the transparency of the implicit phases of career development [39]. Crucially, the process of professional development—the cornerstone of competitive advantage, does not have to be maintained as tacit knowledge that is difficult to duplicate, replace, or transfer; rather, it can and must be redefined and operationalized [40]. The trajectory is intended to offer a comprehensive explanation, exemplification, and description of the way in which T&I teachers incorporate their three sub-roles, culminating in the main role, into a cohesive trajectory that other T&I teachers participating in career development can apply to their own practice. The trajectory's value lies in highlighting the process that often remain implicit or taken for granted in the professional lives of T&I teachers, offering a nuanced understanding of their career development.

## 9 Implications and suggestions

Drawing on the DSMRI, this article has presented a TITRI trajectory as a conceptual model for understanding T&I teachers' career trajectories. The model's three stages are described with rich qualitative data from three case studies. By using multiple case studies, the article provides a conceptual foundation for other T&I teachers and thus generate insight into the nature of career trajectory, contributing to both T&I teacher and other practice-oriented discipline teachers' sustainable development.

The trajectory presented in this article has several practical implications for T&I teachers and policymakers. Firstly, it emphasizes the importance of recognizing the area between the three stages as the area for change [34,41,42], both individually and institutionally. This can help T&I teachers become more aware of their professional development and motivation to progress, while policymakers can optimize their efforts to support the sustained progress along the trajectory. Secondly, the trajectory recognizes the importance of higher education and sociocultural context in T&I professional development, which provides an opportunity for policymakers to investigate the interplay between context and experience, and thus fostering agency in T&I teachers [35]. Thirdly, the trajectory can be personalized, indicating there might be no predetermined optimal stage for individual T&I teachers. Instead, self-assessment [43] is crucial for T&I teachers to recognize their stage of sustainable development within a certain context, and for policymakers to analyze the nature of T&I teachers’ professional development before making any important policy decisions.

The TITRI trajectory may be helpful in several ways. For example, it can be used to inform the design of T&I teacher professional development programs in China as the trajectory highlights the importance of different stages and can be used to tailor professional opportunities and address the needs of teachers at different phases of their careers. Furthermore, it can be used to inform curriculum design for T&I teacher education programs. Since most of the newly T&I teachers are graduates of T&I degree programs, by understanding the trajectory, T&I teacher education programs can be designed to address the needs of pre-service teachers before they embark on their T&I teacher trajectory. The trajectory might also inform further research on the sustainable development of T&I teachers. For example, future research could explore the factors that influence movement between different stages of the trajectory, the impact of different sustainable development interventions on identity development, and the role of context in shaping the role identity trajectory. These findings help educators and policy makers gain insights into in-service T&I teachers’ patterns of professional development, which, in turn, allow them to map out guidance for adequately modeling teacher professional growth and development in teacher preparation courses in higher education institutions.

The limitations of the study should be noted. The size and scope of the sample make it hard to generalize the research findings to other countries or settings without further validation. In addition, as this study only presents a snapshot of T&I teachers' sustainable development by asking them to retell the past, and discuss the present and future plans, without gaining longitudinal data, it does not fully capture the dynamic evolution of career trajectories over time. It also leaves open questions about the potential endpoint of T&I teachers’ professional development or whether the trajectory should be seen cyclical or one-directional. Yet, the TITRI trajectory is effective in promoting reflection and discussion regarding the professional role identity development of T&I teachers.

## Ethics statement

The study involving human participants was reviewed and approved by the Committee on the Use of Human and Animal Subjects in Teaching and Research (HASC) of Hong Kong Baptist University where the first author completed her EdD thesis (which the current manuscript is based on) (Approval Number: REC/Student/19-20/0545). The participants provided their written informed consent to participate in this study.

## Data availability statement

Data will be made available on request.

## CRediT authorship contribution statement

**Bacui Chen:** Writing – review & editing, Writing – original draft, Project administration, Methodology, Investigation, Formal analysis, Data curation, Conceptualization. **Jing Huang:** Writing – review & editing, Validation, Supervision, Resources, Formal analysis, Conceptualization.

## Declaration of competing interest

The authors declare that they have no known competing financial interests or personal relationships that could have appeared to influence the work reported in this paper.
