# Supplementary material for: Becoming and being a translation and interpreting teacher in China: A sustainable role identity trajectory
Source: Heliyon. 2024 Aug 8;10(16):e36013. doi: 10.1016/j.heliyon.2024.e36013 (PMC11366871; doi:10.1016/j.heliyon.2024.e36013)
Supplement: Multimedia component 1 [file mmc1.docx]

**Appendix 1 Initial Interview Guide**

Q1: I am interested in your experience as a T&I teacher. Would you please tell me about when and how you became a T&I teacher?

Q2: What gives you the most satisfaction as a T&I teacher as a job? And what gives you the most frustration?

Q3: What were the most meaningful experiences you had as a T&I trainer/educator, researcher and practitioner respectively?

Q4: Are you satisfied with your T&I training/educating, research and practice now? Why and why not?

Q5: What do you think of the relationship between training and research? Between research practice and between practice and training?

Q5: What are the major difficulties for you to teach and do research and do translation at the same time?

Q6: How do these difficulties affect your views and attitudes towards being a T&I teacher?

Q7: What is your plan for training, research and practice respectively?

Q8: What is your career plan as a T&I teacher?
